# Explore sirtuin family: SIRT5 as a ticket for the entrance of the TBK1–RelA axis to skeletal muscle rejuvenation

**DOI:** 10.1093/lifemedi/lnaf034

**Published:** 2025-10-13

**Authors:** Yifan Ju, Yichen Zhang, Wei Yu

**Affiliations:** State Key Laboratory of Genetics and Development of Complex Phenotypes, School of Life Sciences, Zhongshan Hospital, Fudan University, Shanghai 200438, China; State Key Laboratory of Genetics and Development of Complex Phenotypes, School of Life Sciences, Zhongshan Hospital, Fudan University, Shanghai 200438, China; State Key Laboratory of Genetics and Development of Complex Phenotypes, School of Life Sciences, Zhongshan Hospital, Fudan University, Shanghai 200438, China

As defined by the World Health Organization, aging is a chronic and long-term process characterized by the gradual accumulation of molecular and cellular damage over time, ultimately leading to the decline in the organism’s viability and an increased risk of disease. Aging results from the complex interplay of multiple factors, and the pursuit of longevity remains a challenging and enduring endeavor for humanity.

The sirtuin family, renowned for their deacetylase activity and implicated in longevity, was discovered in the late 20th century. Among the seven reported sirtuin family members, SIRT1 has been the most extensively studied and is considered as a representative member due to its critical role in anti-aging mechanisms. SIRT1 exerts its anti-aging effects through multiple pathways: (1) anti-inflammatory action by suppressing TNF-α and NF-κB signaling; (2) metabolic regulation via AMPK pathway activation to modulate energy homeostasis; (3) mitochondrial stabilization through deacetylation of peroxisome proliferator-activated receptor coactivator 1α (PGC1α), which enhances fatty acid oxidation while suppressing reactive oxygen species (ROS) production. Notably, recent studies have identified that acetylation at the K408 site of SIRT1 dynamically regulates its deacetylase activity, thereby expanding its function. Specifically, SIRT1 K408Q knock-in mice exhibited a protective phenotype against dextran sulfate sodium (DSS)-induced colitis, highlighting the significance of SIRT1 in modulating intestinal inflammatory responses [1]. SIRT2, the sole family member predominantly localized in the cytoplasm, directly participates in cell cycle regulation (via deacetylase activity to modulate mitosis-associated proteins) and cytoskeletal stabilization. In Charcot-Marie-Tooth disease (CMT), a hereditary peripheral neuropathy, mutant glycyl-tRNA synthetase (GARS) loses its binding capacity to SIRT2, leading to reduced α-tubulin acetylation levels. This renders peripheral neurons more susceptible to mechanical breakage, ultimately driving functional degeneration and injury. Notably, studies demonstrate that *Drosophila* with GARS mutations and SIRT2 knockdown exhibit enhanced motor performance and extended lifespan, indicating SIRT2’s potential as a therapeutic target in neuropathology [2]. In aging-related cardiovascular diseases, activation of the SIRT2–LKB1–AMPK signaling axis ameliorates cardiac hypertrophy in murine models [3], while the SIRT2–p66^shc^–mROS axis improves vasomotor dysfunction (e.g. impaired vascular contraction-relaxation) in aged mice [4].

SIRT5 research has emerged relatively late compared to other sirtuins. Localized predominantly in mitochondria, SIRT5 is distinguished by its versatile enzymatic activities, including not only classical deacetylase activity but also desuccinylase, demalonylase, and deglutarylase functions, though studies on these non-canonical modifications remain limited. Protein succinylation, first reported in 2011 by Professor Yingming Zhao’s team, is associated with mitochondrial energy metabolism and pathologies linked to metabolic dysregulation. SIRT5-mediated desuccinylation of aldehyde dehydrogenase 2 (ALDH2) enhances its enzymatic activity, thereby mitigating mitochondrial oxidative stress, reducing pro-inflammatory cytokine levels, and alleviating liver injury [5]. In metabolic diseases, SIRT5 plays a regulatory role in cardiac lipotoxicity and the progression of diabetic cardiac hypertrophy. Studies using high-fat diet/streptozotocin (HFD/STZ)-treated wild-type and SIRT5 knockout mice demonstrated that SIRT5 deficiency sustains succinylation at the K424 site of carnitine palmitoyltransferase 2 (CPT2), impairing fatty acid oxidation and promoting cardiac lipotoxic accumulation [6].

Previous studies on SIRT5-mediated desuccinylation have predominantly focused on its roles in diverse pathologies, with research models limited to cellular and murine systems, remaining insufficient for clinical applications. Recently Guang-Hui Liu’s team has published groundbreaking work in *Nature Metabolism* investigating SIRT5’s anti-aging effects in primate skeletal muscle [7]. This study employed a rigorously structured methodology: (i) Conceptually, the research was initiated at the primate population level by selecting high-functioning cohorts, with skeletal muscle serving as the primary investigative platform. (ii) Experimentally, the team established a comprehensive analytical framework integrating multi-omics approaches (phenotypic, proteomic, and transcriptomic analyses) to identify and prioritize candidate factors, ultimately elucidating the mechanistic role of the SIRT5–TBK1–RelA signaling axis in skeletal muscle aging. (iii) Translationally, the study validated therapeutic potential by overexpressing SIRT5 via adeno-associated virus (AAV)-mediated delivery in murine models, demonstrating significant attenuation of aging-related phenotypes (Fig. 1).

**Figure 1.**
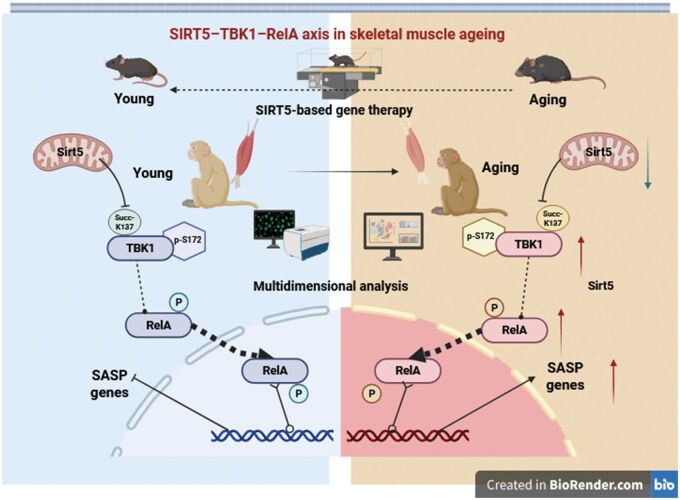
The mechanism of SIRT5–TBK1–RelA in reversing skeletal muscle aging and applications in aging mice.

Skeletal muscle aging represents a pivotal contributor to organismal aging, driving declines in muscle mass and functional capacity while causing some aging-related pathologies such as sarcopenia. Mitochondrial dysfunction marked by diminished oxidative capacity and subsequent dysregulation of cellular energy metabolism serves as a primary driver of skeletal muscle senescence. In addition, extracellular matrix deposition elevates fibrotic remodeling, and the decline in endogenous skeletal muscle stem cells (satellite cells, SCs) impairs tissue self-renewal capacity, collectively exacerbating the condition. Current therapeutic strategies predominantly focus on pharmacological restoration of mitochondrial function or SCs transplantation to enhance regenerative potential, while systematic research into biomarkers and upstream drivers of skeletal ­muscle aging remains underexplored.

In this research, the authors conducted a multidimensional comparative analysis of skeletal muscle samples from young and old, male and female cynomolgus monkeys. They systematically identified biomarker alterations during non-human primate (NHP) skeletal muscle aging across tissue, cellular, and subcellular levels. Comprehensive transcriptomic and quantitative proteomic analyses revealed elevated inflammatory signaling as a hallmark of the skeletal muscle aging. Based on observed downregulation of SIRT5 expression in aged NHP and human skeletal muscle tissues, the authors hypothesized a regulatory role for SIRT5 in primate skeletal muscle aging. This hypothesis was experimentally validated in myotubes that were differentiated from CRISPR-Cas9-mediated *SIRT5^−/−^* human embryonic stem cells.

To further elucidate the mechanism by which SIRT5 ­modulates myotube senescence, the authors employed co-immunoprecipitation (Co-IP) and mass spectrometry in NHP skeletal muscle lysates, revealing an interaction between SIRT5 and TBK1 (a protein implicated in inflammatory regulation) in mitochondria. Based on this discovery, the authors hypothesized that SIRT5 mediates desuccinylation of the TBK1. This hypothesis was confirmed by SIRT5 depletion, desuccinylase-inactive mutant SIRT5-H158Y, and the mutation of TBK1 succinylation site. At the same time, SIRT5 was shown to desuccinylate TBK1 at Lys137 (K137), a modification that subsequently modulates phosphorylation at Ser172 (S172) of TBK1. Importantly, under SIRT5 knockout conditions, TBK1 knockdown reduced RelA phosphorylation levels and attenuated senescent phenotypes, collectively defining the SIRT5–TBK1–RelA axis as the effector to counteract human myotubes (hMyotubes) aging.

Finally, the authors delivered wild-type SIRT5 or its mutant (SIRT5-H158Y) via lentiviral vectors into SIRT5-deficient human myotubes, demonstrating that WT SIRT5 rescued senescence-associated phenotypes. Given the observed decline in SIRT5 expression in aged murine skeletal muscle, the authors administered SIRT5 via lentiviral delivery to aged male mice. This SIRT5-based gene therapy significantly attenuated age-related muscular dysfunction, thereby proposing a strategy for its therapeutic potential in combating skeletal muscle aging.

This study pioneers the use of NHP (cynomolgus monkey) skeletal muscle tissues from both genders to systematically characterize primate skeletal muscle aging phenotypes and identify inflammation as a key biomarker. The authors described SIRT5 as a driver of skeletal muscle aging, a finding subsequently validated in human myotube models. While SIRT5 is recognized for its deacetylase activity and multiple roles in metabolic regulation, this work provides the first *in vivo* evidence of its central role in primate skeletal muscle senescence. Although prior studies have reported SIRT5-mediated desuccinylation of TBK1 in modulating TBK1 activity and macrophage inflammatory responses [8], the discovery that SIRT5 desuccinylates TBK1 at Lys137 (K137) to influence skeletal muscle aging ­highlights the complexity of SIRT5’s enzymatic functions. This finding further implies potential tissue-specific functional divergence within the sirtuin family.

This study provides a theoretical foundation for developing skeletal muscle aging interventions while highlighting critical avenues for future exploration. First, the identification of aging biomarkers and their drivers using NHP models enhances clinical relevance and establishes a robust framework for understanding human aging. Second, the research from mechanistic validation in cynomolgus monkeys and human cellular models to therapeutic testing in aged mice demonstrates evolutionary conservation of the SIRT5–TBK1–RelA axis across species. However, while this axis is validated in skeletal muscle senescence, aging is a systemic process involving multifactorial interactions, and whether its modulation confers synergistic benefits in other tissues remains to be investigated. Beyond gene therapy, this discovery suggests a promising strategy for small molecule therapeutics targeting the members in the pathway.

In summary, as pivotal regulatory factors involving diverse physiological and biochemical processes, the sirtuin family plays critical roles in aging, metabolism, and cellular homeostasis. This study recommends SIRT5 as a novel modulator of skeletal muscle aging. By anchoring inflammation as a central biomarker, we identified pronounced succinylation of TBK1 in SIRT5-deficient senescent human myotubes, which triggers activation of the TBK1–RelA inflammatory axis and promotes chronic inflammation. Mechanistically, SIRT5-dependent desuccinylation of TBK1 at Lys137 facilitates its dephosphorylation at Ser172, thereby inactivating downstream NF-κB signaling cascades. This forms the basis of the SIRT5–TBK1–RelA axis working model in skeletal muscle aging. The molecular mechanisms elucidated in primates were translated therapeutically through lentiviral-mediated gene delivery, indicating SIRT5 overexpression ameliorated age-related skeletal muscle dysfunction. These findings propose a potential intervention strategy for mitigating muscle senescence and underscore the therapeutic promise of sirtuin-mediated regulation in combating aging-related pathologies.

## References

[1] Xie L , Li C, Wang C et al Aspirin-mediated acetylation of SIRT1 maintains intestinal immune homeostasis. Adv Sci 2024;11:2306378. 10.1002/advs.202306378 PMC11109641 38482749

[2] Zhao Y , Xie L, Shen C, et al SIRT2-knockdown rescues GARS-induced Charcot-Marie-Tooth neuropathy. Aging Cell 2021; 20: e13391. 34053152 10.1111/acel.13391 PMC8208790

[3] Tang X , Chen X-F, Wang N-Y et al SIRT2 acts as a cardioprotective deacetylase in pathological cardiac hypertrophy. Circulation 2017;136:2051–67. 28947430 10.1161/CIRCULATIONAHA.117.028728 PMC5698109

[4] Zhang Y , Wang X, Li X-K et al Sirtuin 2 deficiency aggravates ageing-induced vascular remodelling in humans and mice. Eur Heart J 2023;44:2746–59. 37377116 10.1093/eurheartj/ehad381 PMC10393077

[5] Yu Q , Zhang J, Li J et al Sirtuin 5-mediated desuccinylation of ALDH2 alleviates mitochondrial oxidative stress following Acetaminophen-induced acute liver injury. Adv Sci 2024;11:2402710. 10.1002/advs.202402710 PMC11497042 39159058

[6] Wu M , Tan J, Cao Z et al Sirt5 improves cardiomyocytes fatty acid metabolism and ameliorates cardiac lipotoxicity in diabetic cardiomyopathy via CPT2 de-succinylation. Redox Biol 2024;73:103184. 38718533 10.1016/j.redox.2024.103184 PMC11091707

[7] Zhao Q , Jing Y, Jiang X et al SIRT5 safeguards against primate skeletal muscle ageing via desuccinylation of TBK1. Nat Metab 2025;7:556–73. 40087407 10.1038/s42255-025-01235-8

[8] Zhang X , Ling C, Xiong Z et al Desuccinylation of TBK1 by SIRT5 regulates inflammatory response of macrophages in sepsis. Cell Rep 2024;43:115060. 39673708 10.1016/j.celrep.2024.115060

